# Preparation of PEDOT/GO, PEDOT/MnO_2_, and PEDOT/GO/MnO_2_ nanocomposites and their application in catalytic degradation of methylene blue

**DOI:** 10.1186/s11671-015-0859-6

**Published:** 2015-03-25

**Authors:** Li Zhang, Ruxangul Jamal, Qin Zhao, Minchao Wang, Tursun Abdiryim

**Affiliations:** Key Laboratory of Oil and Gas Fine Chemicals, Educational Ministry of China, College of Chemistry and Chemical Engineering, Xinjiang University, Shengli Road No.14, Tianshan District, Urumqi, Xinjiang 830046 People’s Republic of China; Key Laboratory of Functional Polymers, Xinjiang University, Shengli Road No.14, Tianshan District, Urumqi, Xinjiang 830046 People’s Republic of China

**Keywords:** Poly(3,4-ethylenedioxythiophene), MnO_2_, Graphene oxide, Composite, Methylene blue

## Abstract

The nanocomposite materials of poly(3,4-ethylenedioxythiophene)/graphene oxide (PEDOT/GO), poly(3,4-ethylenedioxythiophene)/MnO_2_ (PEDOT/MnO_2_), and poly(3, 4-ethylenedioxythiophene)/graphene oxide/MnO_2_ (PEDOT/GO/MnO_2_) were successfully prepared by facile and template-free solution method. The structure and morphology of nanonanocomposites were characterized by Fourier transform infrared spectroscopy (FTIR), ultraviolet–visible absorption spectra (UV–vis), field emission scanning electron microscope (FESEM), X-ray diffraction (XRD), and energy-dispersive X-ray spectroscopy (EDX), respectively. The catalytic activities of nanocomposites were investigated through the degradation processes of methylene blue (MB) solution under dark, UV light, and nature sunlight irradiation, respectively. The results displayed that nanocomposites were successfully synthesized, and PEDOT/GO had higher conjugation length and doped degree than pure PEDOT. However, the introduction of MnO_2_ could lead to the reduction of conjugation length and doped degree in PEDOT/MnO_2_ and PEDOT/GO/MnO_2_ nanocomposites. The field emission scanning electron microscope (FESEM) analysis also showed that both MnO_2_ and GO had some effect on the morphology of nanocomposites. The catalytic activities of pure PEDOT and nanocomposites were in the order of PEDOT/GO/MnO_2_ > PEDOT/MnO_2_ > PEDOT/GO > pure PEDOT. Besides, the catalytic results also showed that the highest degradation efficiency of MB after 7 h occurred in the PEDOT/GO/MnO_2_ composite in three irradiation.

## Background

Dyes, pigments, and their causative compounds are difficult for industrialization since they are highly carcinogenic and undesirable in water as reported. Consequently, it is necessary to remove them from wastewater before discharge. Methylene blue (MB) is a water-soluble thiazine dye, commonly used for dyeing of silk, leather, plastics, and paper. On inhalation, MB can give rise to short periods of rapid or difficult breathing while ingestion through the mouth may cause hypertension and discomfort. To prevent harmful impacts of MB on receiving waters, the degradation of MB is of great importance in water treatment [1,2]. In recent years, catalysts have attracted much attention of scientists to removal of dyes [3,4]. Among the various catalysts, MnO_2_ is considered as one of the most outstanding metal oxides on new catalytic oxidation systems, due to its relative low price, chemical stability, and non-toxic property. By now, many efforts have been made on the application of MnO_2_ in battery materials, supercapacitor, and catalysts [5-10]. However, few studies have attention on the conductive polymers/MnO_2_ composites in water treatment. Among the conducting polymers, poly(3,4-ethylenedioxythiophene) (PEDOT) has become commercially available conducting polymer because of its high conductivity, effective thermal stability, fast charge/discharge ability, low-oxidation potential, and high cycling stability [11-13]. In addition, delocalized conjugated structures of conductive polymers have been proven to arouse a rapid photoinduced charge separation and decrease the charge recombination rate in electron-transfer processes [14,11]. Until now, many chemical methods have been reported for the formation of conducting polymer/MnO_2_ composites. Commonly, monomers are always oxidized to obtain the conducting polymer, and KMnO_4_ is reduced to give MnO_2_. Despite the obvious advantages of simplicity and reproducibility in this synthetic method, the morphology of such composites and the size of MnO_2_ particles are normally hard to control. More interestingly, it has been recently revealed that conducting polymers themselves show redox activity toward KMnO_4_, where KMnO_4_ is always reduced to MnO_2_ [15]. As the redox reactions occur on the surfaces of conducting polymers, MnO_2_ will prefer to adsorb on the surfaces of the conducting polymer. Recently, graphene oxide (GO) has received intensive attention owing to the fascinating mechanical, electrical, thermal, and optical properties. In comparison with other carbon materials, GO has the perfect sp^2^ hybrid carbon nanostructure and various oxygen groups including epoxide, hydroxyl, carbonyl, and carboxyl groups. In addition, the conjugation of GO with semiconductor solid particles results in catalysts with improved charge separation, reduced recombination of the photogenerated electron–hole pairs, increased specific surface area, and an adequate quantity of adsorption sites, which could lead to the enhancement of degradation efficiency of wastewater [16-19]. On account of the abovementioned advantages, the reasonable combination of PEDOT, GO, and MnO_2_ would produce some novel composites with excellent catalytic performance.

Herein, we report the synthesis of binary and ternary nanocomposites of poly(3,4-ethylenedioxythiophene)/graphene oxide (PEDOT/GO), poly(3,4-ethylenedioxythiophene)/MnO_2_ (PEDOT/MnO_2_), and poly(3, 4-ethylenedioxythiophene)/graphene oxide/MnO_2_ (PEDOT/GO/MnO_2_) via facile and template-free solution process. The structural and morphological properties of nanocomposites were investigated by Fourier transform infrared spectroscopy (FTIR), ultraviolet–visible absorption spectra (UV–vis), field emission scanning electron microscope (FESEM), X-ray diffraction, and energy-dispersive X-ray spectroscopy (EDX). Furthermore, nanocomposites were tested as catalysts for removal of MB from aqueous solution under different light sources.

## Methods

### Materials

3,4-Ethylenedioxythiophene (EDOT) was obtained from Shanghai Aladdin Reagent Company (Shanghai, China), and it was purified by distillation under reduced pressure and stored in a refrigerator prior to use. All other chemicals and solvents, including FeCl_3_ · 6H_2_O, potassium permanganate (KMnO_4_), ethanol, and chloroform, were used as received without further purification.

### Preparation of the PEDOT and PEDOT/GO

The procedure for synthesis of PEDOT was as follows: the pure PEDOT polymer was prepared by mixing a 5.0 mL of 14.2 wt.% EDOT alcoholic solution and 20 mL FeCl_3_ · 6H_2_O (2.0 M) aqueous solution. The reaction system was maintained under magnetic stirring for 24 h. Then, the resulting mixture was filtered, washed, and dried at 60°C for 12 h.

The procedure for synthesis of PEDOT/GO composite was as follows: 0.04 g GO was dissolved in 20 mL FeCl_3_ · 6H_2_O (2.0 M) aqueous solution; then, the PEDOT/GO composite was prepared by mixing a 5.0 mL of 14.2 wt.% EDOT alcoholic solution and FeCl_3_ · 6H_2_O aqueous solution. The reaction system was maintained under magnetic stirring for 24 h. Then, the resulting mixture was filtered, washed, and dried at 60°C for 12 h.

### Preparation of PEDOT/MnO_2_ and PEDOT/GO/MnO_2_

The procedure for preparation of PEDOT/MnO_2_ nanocomposites was as follows: the incorporation of MnO_2_ was carried out by soaking 0.08 g PEDOT powders into KMnO_4_ aqueous solution (0.16 g, 10 mM). Then, the reaction system was maintained under magnetic stirring at room temperature for 10 min. The products were further rinsed several times with deionized water and dried under vacuum at 60°C for 24 h. PEDOT/GO/MnO_2_ composite was synthesized by the same method but only used PEDOT/GO powders instead of PEDOT.

### Characterization techniques

The Fourier transform infrared spectra of the samples were measured on a BRUKER EQUINOX-55 Fourier transform infrared spectrometer (Bruker Optics, Billerica, MA, USA) at a resolution of 4 cm^−1^ using the KBr technique. UV–vis spectra of the samples were recorded on a UV-visible spectrophotometer (UV4802, Unico, Dayton, NJ, USA). The XRD patterns have been obtained by using a Bruker AXS D8 diffractometer (Bruker AXS Inc., Madison, WI, USA), and the scan range was 10° to 80°, with monochromatic Cu-Ka radiation source (*λ* = 0.15418 nm). The elemental percentages of samples were measured on energy-dispersive X-ray spectroscopy, which was taken on a Leo1430VP microscope (Carl Zeiss Inc., Oberkochen, Germany) with operating voltage 5 kV. The process of EDX measures were carried out with a pellet which was pressed at 200 MPa and then adhered to copper platens. Morphology and microstructure of the samples were investigated by field emission scanning electron microscope (FESEM Hitachi S-4800, Hitachi Ltd., Chiyoda-ku, Japan).

### Degradation experiments of methylene blue

The catalytic activity of pure PEDOT and nanocomposites were performed using MB dyes as degraded materials in quartz tubes under dark, UV light, and natural sunlight irradiation. Of catalysts (PEDOT), 20 mg was shaken in MB solution (50 mL) and stirred for 7 h. After degradation of dye, the catalysts were removed by centrifugation. FSL MW1-Y15 (Foshan Electrical and Lighting Co., Ltd., Guangdong, China) was used as the irradiation source (*λ* = 254 nm) located in a light infiltrated chamber. The degradation study of PEDOT/GO, PEDOT/MnO_2_, and PEDOT/GO/MnO_2_ nanocomposites was conducted by the same method.

## Results and discussion

### Fourier transform infrared spectroscopy

**Figure 1:**
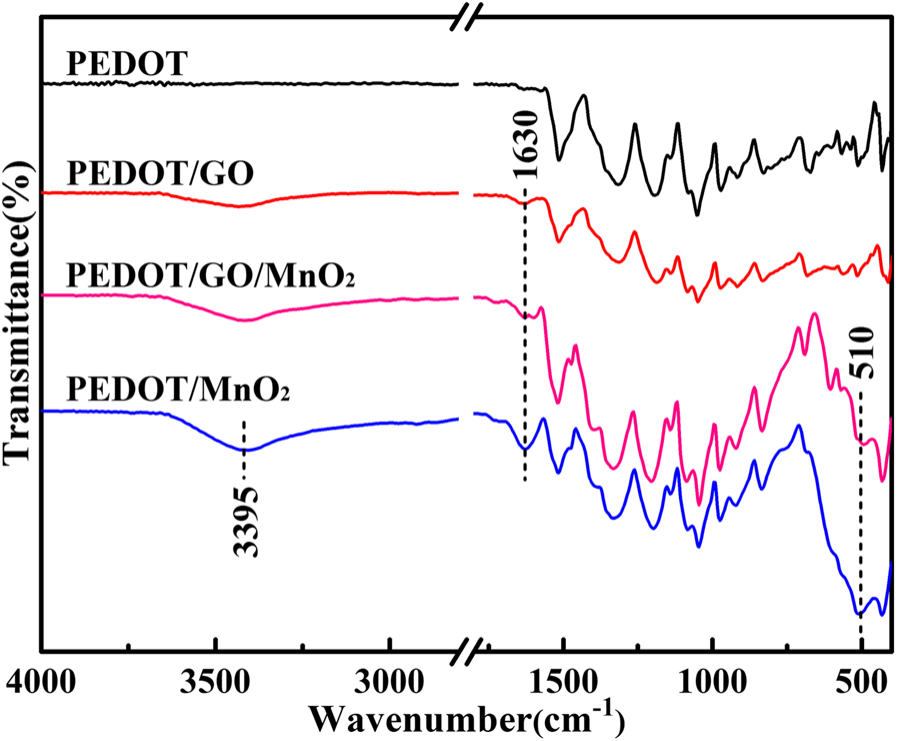
**FTIR spectra of pure PEDOT, PEDOT/GO, PEDOT/MnO**_**2**_ **, and PEDOT/GO/MnO**_**2**_ **nanocomposites.**

Figure 1 shows the FTIR spectra of PEDOT, PEDOT/GO, PEDOT/MnO_2_, and PEDOT/GO/MnO_2_ nanocomposites. As shown in Figure 1, the characteristic absorption bands of the PEDOT and PEDOT/GO are at 1,510, 1,316, 1,176, 1,140, 1,044, 921, 831, and 704 cm^−1^, respectively. The bands of 1,510, 1,316, 1,176, 1,140, and 1,044 cm^−1^ are assigned to the aromatic C = C stretching in benzene ring of polythiophene and the C-O-C bond stretching in ethylenedioxy group, respectively [20]. The presence of C-S-C bond in the thiophene ring is indicated by the bands at 921, 831, and 704 cm^−1^ [21]. Moreover, several absorption bands around 3,395 and 1,630 cm^−1^ are observed in PEDOT/GO, PEDOT/MnO_2_, and PEDOT/GO/MnO_2_ nanocomposites, which can be attributed to stretching and bending vibrations of the -OH group of crystal and adsorbed water molecules, respectively [22]. In addition, the band of Mn − O and Mn − O − Mn vibrations at 510 cm^−1^ is observed in the case of PEDOT/MnO_2_ and PEDOT/GO/MnO_2_ nanocomposites, which supports that the presence of MnO_2_ in nanocomposites [23].

### UV–vis spectra

**Figure 2:**
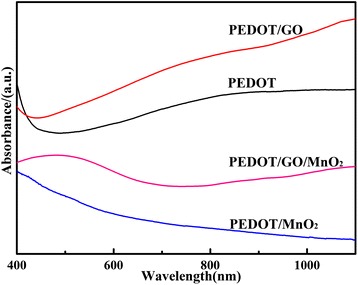
**UV–vis spectra of pure PEDOT, PEDOT/GO, PEDOT/MnO**_**2**_ **, and PEDOT/GO/MnO**_**2**_ **nanocomposites.**

Figure 2 shows the UV–vis absorption spectra of PEDOT, PEDOT/GO, PEDOT/MnO_2_, and PEDOT/GO/MnO_2_ nanocomposites, respectively. As can be seen in Figure 2, pure PEDOT displays a broad absorption band, starting at *ca*.450 nm and extending into the mid IR region, which is very similar to that of previously reported PEDOT [12]. Compared with pure PEDOT, the higher intensity of absorption peaks near 700 to 1,000 nm is observed in the case of PEDOT/GO nanocomposites, which corresponds to the polymer having a longer conjugation length with greater order [12,24]. This result suggests that PEDOT/GO nanocomposites have the higher conjugation length and doped degree than pure PEDOT. However, the introduction of MnO_2_ in PEDOT/MnO_2_ and PEDOT/GO/MnO_2_ nanocomposites will lead to the reduction of conjugation length and doped degree. Further comparison indicates that PEDOT/GO/MnO_2_ has the higher conjugation length and doped degree than that of PEDOT/MnO_2_. Moreover, a broad band between 400 and 700 nm with *λ*_max_ at 500 nm is observed in PEDOT/GO/MnO_2_, which can be attributed to the π-π* transition of the thiophene ring [25].

### Field emission scanning electron microscope

**Figure 3:**
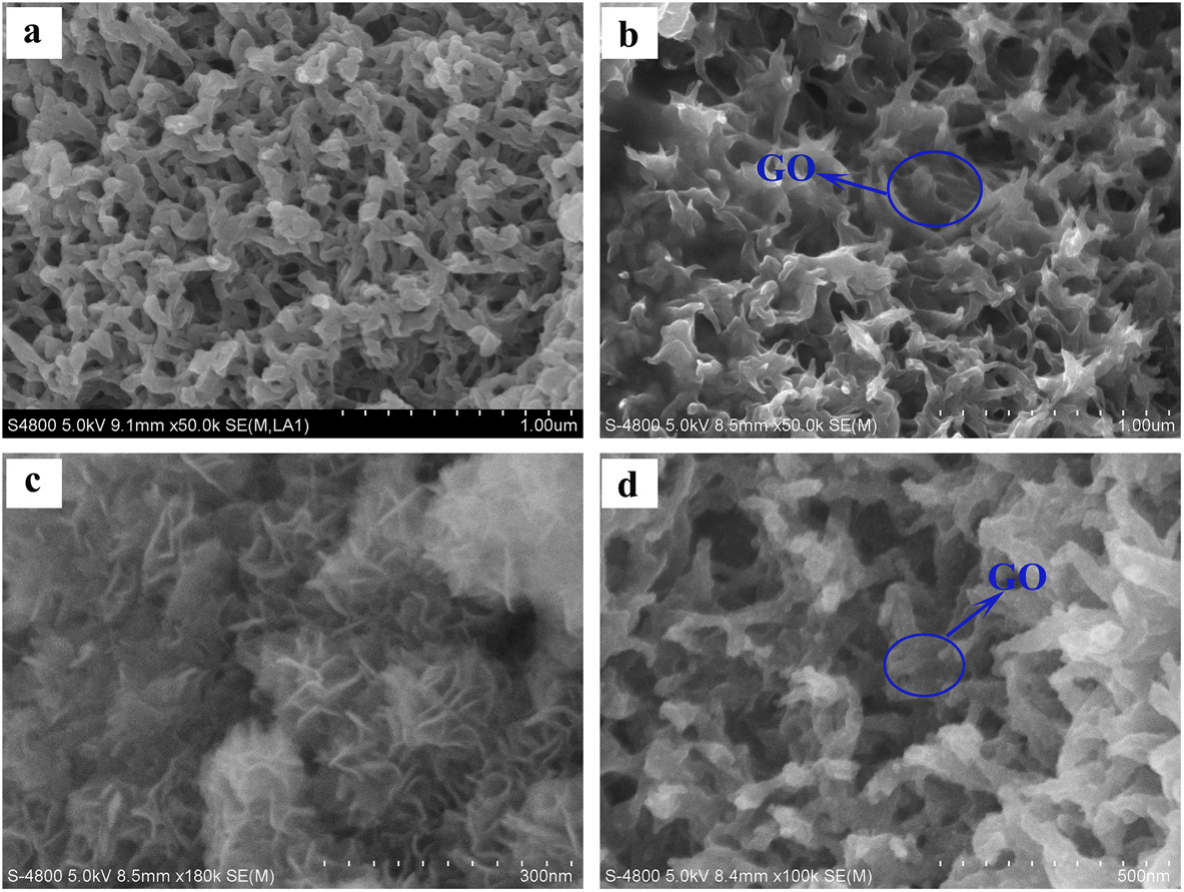
**FESEM images of (a) pure PEDOT, (b) PEDOT/GO, (c) PEDOT/MnO**_**2**_ **, and (d) PEDOT/GO/MnO**_**2**_ **nanocomposites.**

Figure 3 shows the FESEM images of PEDOT, PEDOT/GO, PEDOT/MnO_2_, and PEDOT/GO/MnO_2_ nanocomposites, respectively. As depicted in Figure 3a,b,c,d, pure PEDOT exhibits coral-like morphology and PEDOT/MnO_2_ shows flower-like morphology. After the formation of PEDOT/GO and PEDOT/GO/MnO_2_, the presence of thin-layered GO can be seen in the nanocomposites and GO makes solely coral-like morphology of PEDOT connected with each other to form the porous structure. In addition, many flocculent structures are clearly observed on the surface of PEDOT/GO and PEDOT/GO/MnO_2_ nanocomposites, suggesting the strong interactions between PEDOT and GO in the nanocomposite. The above results show that both MnO_2_ and GO have some effect on the morphology of nanocomposites.

### X-ray diffraction

**Figure 4:**
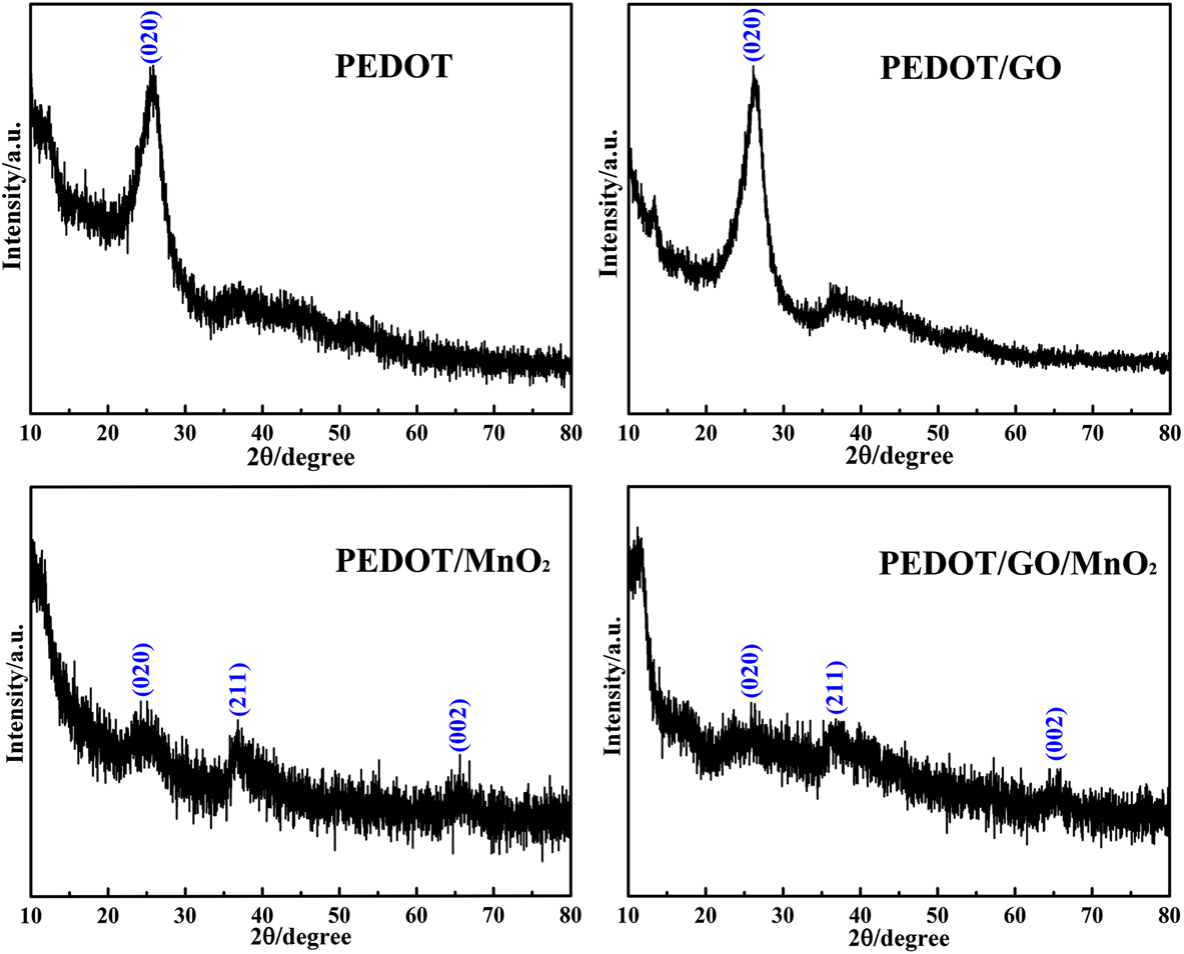
**XRD patterns of pure PEDOT, PEDOT/GO, PEDOT/MnO**_**2**_ **, and PEDOT/GO/MnO**_**2**_ **nanocomposites.**

Figure 4 shows the XRD patterns of PEDOT, PEDOT/GO, PEDOT/MnO_2_, and PEDOT/GO/MnO_2_ nanocomposites, respectively. As shown in Figure 4, the pure PEDOT and PEDOT/GO display broad diffraction peaks with low intensity at 2*θ* = 26°, which can be attributed to the intermolecular spacing of polymer backbone or assigned to the (020) reflection [26]. Besides, two diffraction peaks at 2*θ* = 37° (211) and 66° (002) indexed to MnO_2_ (JCPDS No. 44–0141) are observed in the case of PEDOT/MnO_2_ and PEDOT/GO/MnO_2_ nanocomposites, suggesting the successful incorporation of MnO_2_ in nanocomposites [27,28]. However, the XRD pattern of MnO_2_ in the nanocomposites showed broad and low peaks, revealing a poorly crystalline phase of MnO_2_. Besides, the diffraction peaks from GO are difficult to detect in the PEDOT/GO and PEDOT/GO/MnO_2_ nanocomposites, which may be due to a low mass ratio.

### Energy-dispersive X-ray spectroscopy

**Figure 5:**
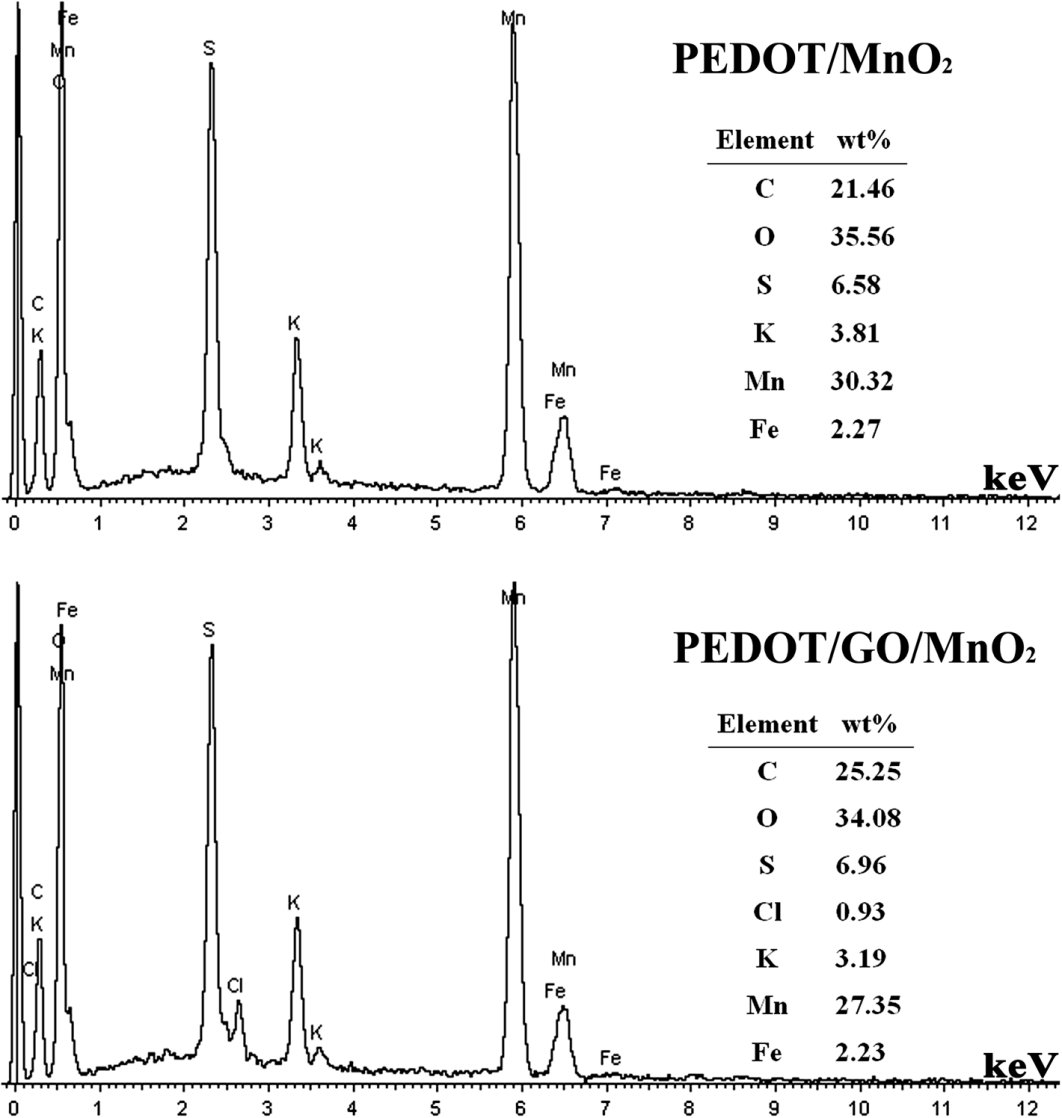
**EDX spectra of PEDOT/MnO**_**2**_ **and PEDOT/GO/MnO**_**2**_ **nanocomposites.**

To study the element percentage of Mn in PEDOT/MnO_2_ and PEDOT/GO/MnO_2_ nanocomposites, EDX results of nanocomposites are presented in Figure 5. The results show that the percentage of Mn in PEDOT/MnO_2_ and PEDOT/GO/MnO_2_ nanocomposites are 30.32 wt.% and 27.35 wt.%, respectively. Furthermore, the element of Cl and Fe are observed in nanocomposites, which should be resulted from the doping agent of FeC1_4_^−^.

### Degradation experiments of methylene blue

The degradation of MB dyes was performed in the presence of pure PEDOT, PEDOT/GO, PEDOT/MnO_2_, and PEDOT/GO/MnO_2_ nanocomposites as catalysts under dark, UV light, and sunlight sources, respectively. The degradation efficiency of samples (*R*, %) was calculated by the following equation: *R* = [*C*_o_ − *C*/*C*_o_] (where *C*_o_ represents the initial concentration of dye, *C* represents the concentration of dye after a certain irradiation time, respectively).

**Figure 6:**
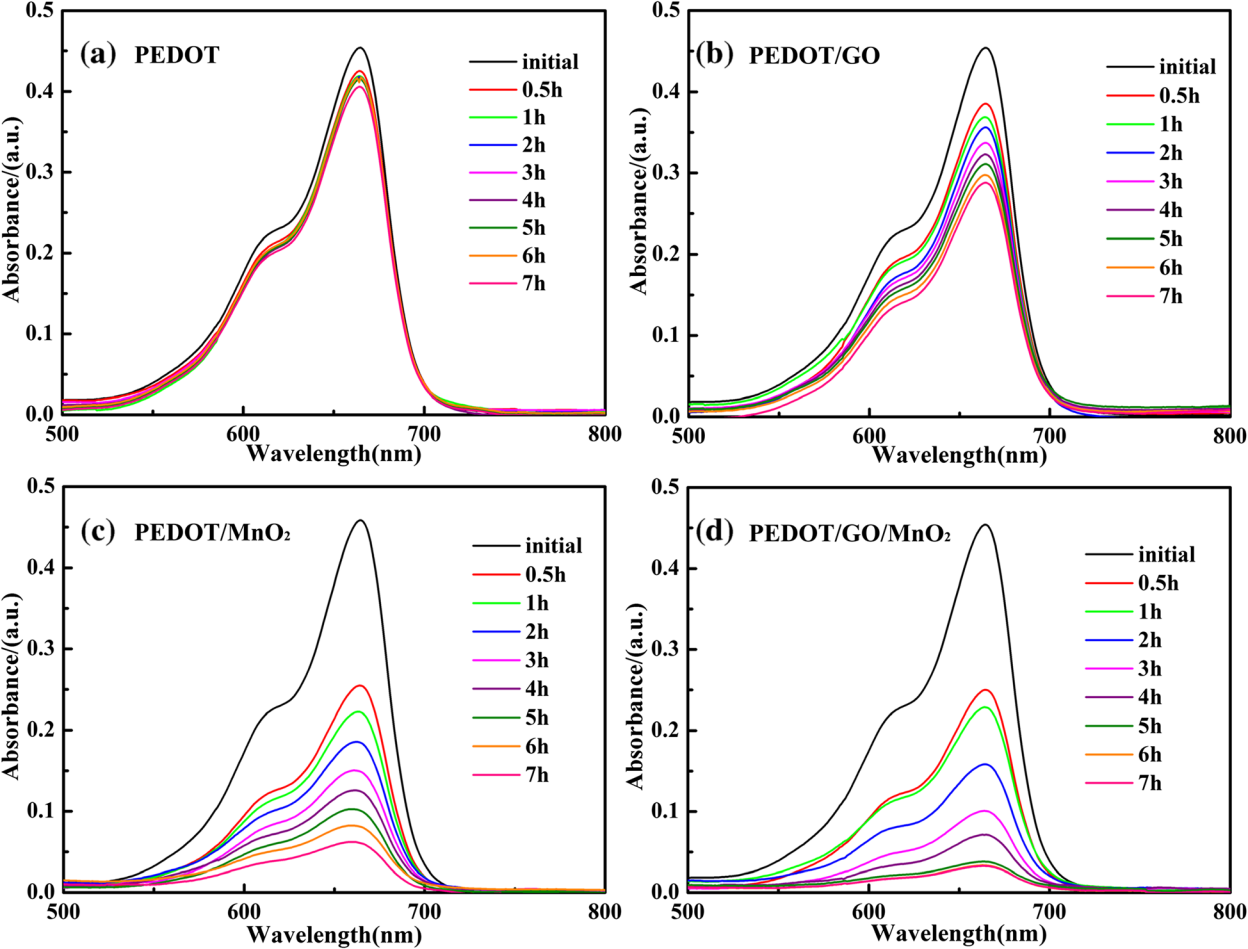
**UV–vis absorption spectra of MB dye under dark. (a)** PEDOT. **(b)** PEDOT/GO. **(c)** PEDOT/MnO_2_. **(d)** PEDOT/GO/MnO_2_.

**Figure 7:**
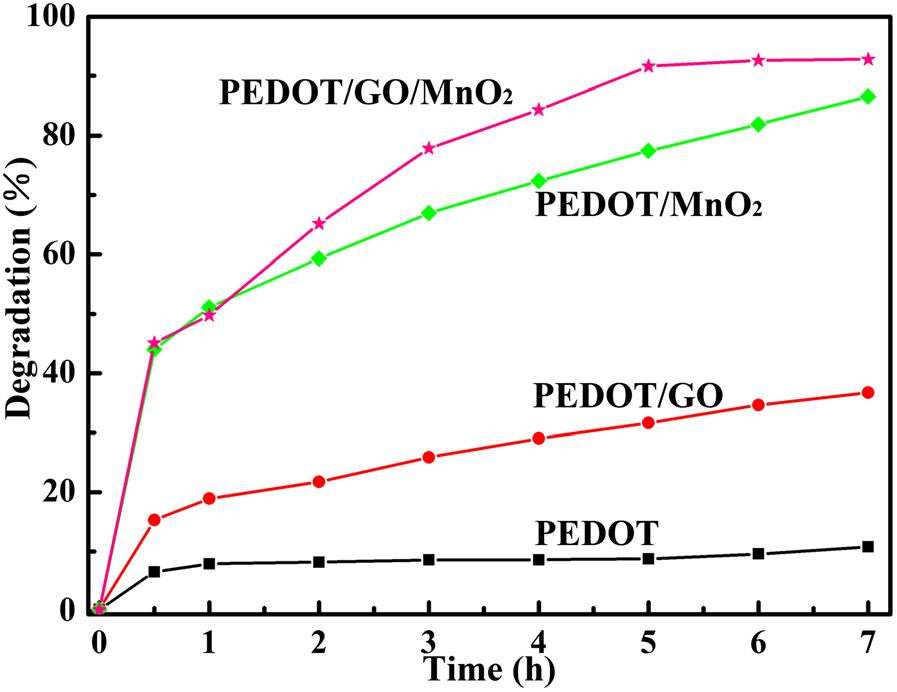
**Time profiles of MB degradation with pure PEDOT, PEDOT/GO, PEDOT/MnO**_**2**_ **, and PEDOT/GO/MnO**_**2**_ **nanocomposites under dark.**

Figure 6 shows the degradation spectra of MB in the presence of pure PEDOT, PEDOT/GO, PEDOT/MnO_2_, and PEDOT/GO/MnO_2_ nanocomposites under dark, respectively. The time profiles of MB degradation with the pure PEDOT, PEDOT/GO, PEDOT/MnO_2_, and PEDOT/GO/ MnO_2_ nanocomposites under dark were shown in Figure 7. As can be seen in Figures 6 and 7, the degradation efficiencies of the MB dye by PEDOT, PEDOT/GO, PEDOT/MnO_2_, and PEDOT/GO/MnO_2_ nanocomposites are 9.9%, 36.5%, 86.2%, and 92.7% under dark, respectively. According to the SEM analysis, the pure PEDOT exhibits coral-like morphology with a smaller pore size. With the addition of GO, the PEDOT/GO displays flocculent structures with a larger pore size, which is beneficial for the physical adsorption of MB molecule. Furthermore, due to formation of π-π stacking between MB and aromatic regions of the graphene oxide in the PEDOT/GO composite, the physical adsorption of MB molecules can be promoted [16,29]. It should be noted that specific area of pure PEDOT is higher than PEDOT/GO, and the high specific area can enhance the adsorbability of MB by electrostatic interaction between the polymer chains and MB molecules [30]. However, compared with the pure PEDOT (9.9%), the higher degradation efficiency of MB solution occurred in PEDOT/GO (36.5%) composite. This result shows that the presence of GO in PEDOT/GO composite can be the main factor for improving the physical adsorption of MB molecules, suggesting that the GO has higher adsorbability for MB than that of PEDOT. Moreover, the degradation of MB by pure PEDOT and PEDOT/GO composite is physical adsorptive removal of MB molecules. However, the higher degradation efficiencies of MB solution are observed in the case of PEDOT/MnO_2_ (86.2%) and PEDOT/GO/MnO_2_ (92.7%) nanocomposites than that of pure PEDOT (9.9%) and PEDOT/GO (36.5%) nanocomposite. As shown in Figure 3, a lot of wrinkles are observed on the flower-like morphology of PEDOT/MnO_2_, and PEDOT/GO/MnO_2_ displays flocculent structures with a larger pore size than that of PEDOT/GO. Based on the discussion about the effect of morphology of pure PEDOT and PEDOT/GO on the physical adsorptive removal of MB molecules, it is clear that the flower-like morphology of PEDOT/MnO_2_ and flocculent structure of PEDOT/GO/MnO_2_ are beneficial for the physical adsorption of MB molecule on the surface of nanocomposites. However, if the physical adsorptive removal of MB molecules is the decisive factor for enhancing the degradation efficiency of MB solution, the degradation efficiency of MB is impossible to be 86.2% and 92.7% for PEDOT/MnO_2_ and PEDOT/GO/MnO_2_, respectively. Thus, the presence of MnO_2_ in both of PEDOT/MnO_2_ and PEDOT/GO/MnO_2_ composites will be the most important factor for determining the degradation efficiency of MB. Previous studies show that the MnO_2_ is one of the effective catalysts for degradation of MB, which can be attributed to the formation of hydrogen bonding between the hydroxyl groups on the surface of MnO_2_ and the nitrogen atoms of MB [31]. Therefore, it can be concluded that the presence of MnO_2_ in PEDOT/MnO_2_ and PEDOT/GO/MnO_2_ plays an important role on the enhancement of degradation of MB under dark.

**Figure 8:**
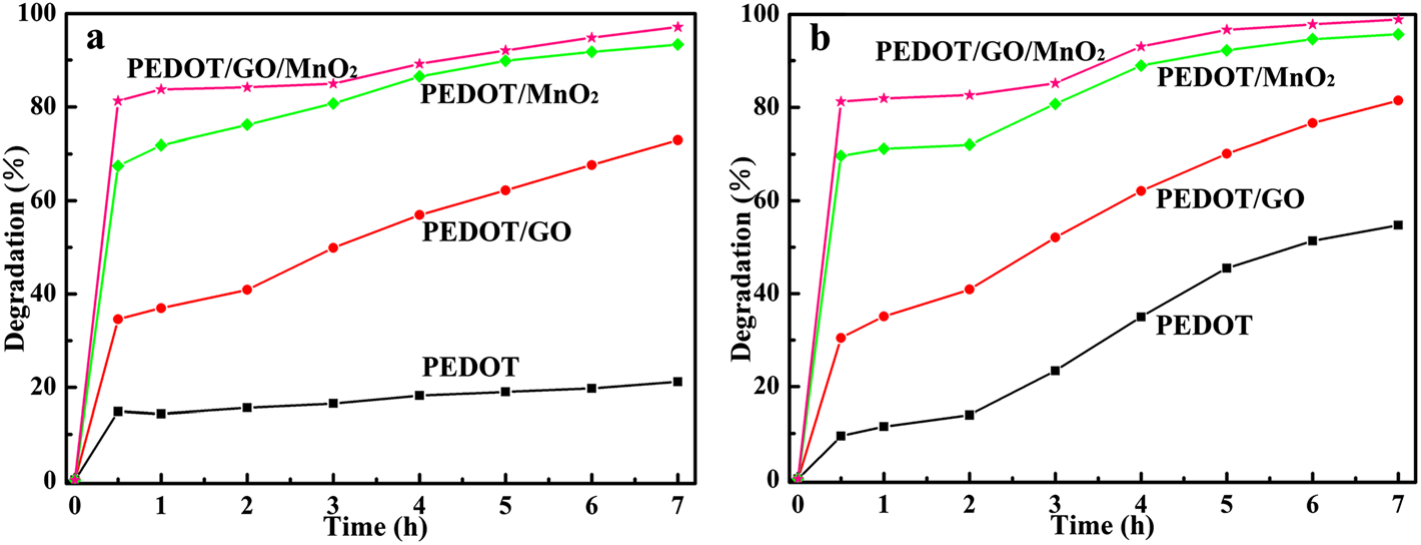
**The degradation of MB with pure PEDOTand nanocomposites.** Under UV light **(a)** and sunlight **(b)**.

Figure 8a,b shows time profiles of MB degradation with the pure PEDOT, PEDOT/GO, PEDOT/MnO_2_, and PEDOT/GO/MnO_2_ nanocomposites under UV light and sunlight sources, respectively. As can be seen in Figure 8a,b, the degradation efficiency of the MB in the presence of pure PEDOT, PEDOT/GO, PEDOT/MnO_2_, and PEDOT/GO/MnO_2_ nanocomposites are 21.2%, 72.9%, 93.3%, and 97.1% under UV light and 54.7%, 81.4%, 95.7%, and 98.9 % under natural sunlight, respectively. One can clearly see that the degradation efficiency of the MB in the presence of pure PEDOT under natural sunlight (54.7%) was much higher than under the UV light (21.2%). This result may be due to the pure PEDOT that can absorb the visible light and produces an electron (e^−^), which will promote the charge separation and the formation of oxyradicals (O_2_, HO_2_, ·OH) [32]. Consequently, the high amounts of oxyradicals result in high degradation efficiency of MB under natural sunlight. Besides, it also indicates that the degradation efficiency of the MB in the presence of PEDOT/GO, PEDOT/MnO_2_, and PEDOT/GO/MnO_2_ nanocomposites under natural sunlight were much higher than that of nanocomposites under the UV light. Furthermore, GO as an excellent electron acceptor and transporter could reduce the recombination of charge carriers and enhance the catalytic activity [33,34], and this will be the main reason for higher degradation efficiency of the MB for PEDOT/GO than that of pure PEDOT under UV light and natural sunlight illumination. A further comparison displays that the highest degradation efficiency of the MB is observed in the case of PEDOT/GO/MnO_2_ composite under dark, UV light, and natural sunlight, respectively, suggesting that the degradation efficiency of MB solution influenced by the synergic effects of PEDOT, GO, and MnO_2_.

## Conclusions

In this work, the composite materials of PEDOT/GO, PEDOT/MnO_2_, and PEDOT/GO/MnO_2_ were successfully synthesized by facile and template-free solution method. The results showed that nanocomposites were successfully prepared. Moreover, PEDOT/GO had higher conjugation length and doped degree than pure PEDOT, which may be due to the positive effect of GO on the degree of polymerization of PEDOT. And the results also displayed that both GO and MnO_2_ had some effect on the morphology of nanocomposites. Due to pure PEDOT can absorb the visible light and promote the formation of oxyradicals, the degradation efficiency of the MB in the presence of PEDOT and nanocomposites under natural sunlight were much higher than under UV light. Although the physical adsorption of nanocomposites has some effect on the degradation of the MB, the catalysis effect is the main factor to enhance the degradation efficiency of the MB. Among pure PEDOT and nanocomposites, the highest degradation efficiency of MB was observed in the case of PEDOT/GO/MnO_2_ nanocomposites under different light sources. This phenomenon can be attributed to the hydrogen bonding between surface hydroxyl groups of MnO_2_ and the nitrogen atoms of MB. Another reason may be due to the formation of π-π stacking between MB and aromatic regions of the graphene oxide, and graphene oxide as an excellent electron acceptor and transporter could reduce the recombination of charge carriers and enhance the catalytic activity. That is to say, the synergetic effects between PEDOT and MnO_2_ as well as GO would be combined together to affect the catalytic activity of nanocomposites.
